# Tissue Nanotransfection Silicon Chip and Related Electroporation-Based Technologies for In Vivo Tissue Reprogramming

**DOI:** 10.3390/nano14020217

**Published:** 2024-01-19

**Authors:** Yi Xuan, Cong Wang, Subhadip Ghatak, Chandan K. Sen

**Affiliations:** McGowan Institute for Regenerative Medicine, Department of Surgery, University of Pittsburgh School of Medicine, Pittsburgh, PA 15219, USA

**Keywords:** tissue nanotransfection, electroporation, gene transfer, cell reprogramming

## Abstract

Tissue nanotransfection (TNT), a cutting-edge technique of in vivo gene therapy, has gained substantial attention in various applications ranging from in vivo tissue reprogramming in regenerative medicine, and wound healing to cancer treatment. This technique harnesses the advancements in the semiconductor processes, facilitating the integration of conventional transdermal gene delivery methods—nanoelectroporation and microneedle technologies. TNT silicon chips have demonstrated considerable promise in reprogramming fibroblast cells of skin in vivo into vascular or neural cells in preclinical studies to assist in the recovery of injured limbs and damaged brain tissue. More recently, the application of TNT chips has been extended to the area of exosomes, which are vital for intracellular communication to track their functionality during the wound healing process. In this review, we provide an in-depth examination of the design, fabrication, and applications of TNT silicon chips, alongside a critical analysis of the electroporation-based gene transfer mechanisms. Additionally, the review discussed the existing limitations and challenges in the current technique, which may project future trajectories in the landscape of gene therapy. Through this exploration, the review aims to shed light on the prospects of TNT in the broader context of gene therapy and tissue regeneration.

## 1. Introduction

**Figure 1:**
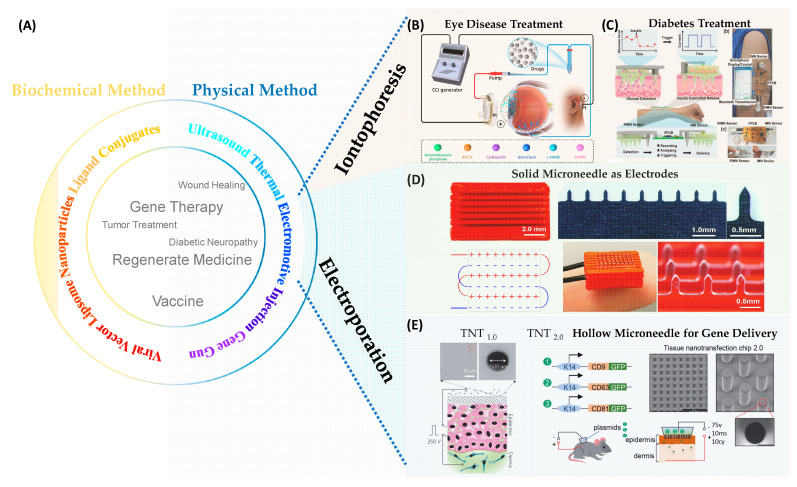
Overview of In Vivo Gene Transfer Methods. (**A**) Gene transfer methods and applications. Yellow part: biochemical gene transfer methods. Blue part: physical gene transfer methods. (**B**) Illustration of iontophoresis treatment in eye disease. Figure reproduced from [11] under terms of the CC-BY license. Copyright 2023, The Author(s), published by Informa UK Limited, trading as Taylor & Francis Group. (**C**) Illustration of iontophoresis in diabetes management. Figure reproduced from [14] under terms of the CC-BY license. Copyright 2021, The Authors, Advanced Science published by Wiley-VCH GmbH. (**D**) Microelectrode-based Electroporation. Figure adapted from [8] under terms of the CC-BY license. Copyright, 2021, the Author(s), Published by PNAS. (**E**) Tissue nanotransfection (TNT) for in vivo gene transfer. Left figure: The first generation of TNT silicon devices (TNT_1.0_). The figure is adapted from [10] with permission. Copyright 2017, Springer Nature Limited. Right figure: The second generation of TNT silicon devices (TNT_2.0_). The figure is adapted from [15] with permission. Copyright, 2020 American Chemical Society. The figures in (**E**) are not subject to the open access terms of this article and may not be reproduced without additional permission from the original copyright holder.

Gene transfer, a key technique in gene therapy, offers transformative potential for disease treatment and vaccine technology [1,2,3,4,5,6,7,8,9,10,11]. In the process of developing clinical gene transfer techniques, it is essential to evaluate the gene expression efficacy and safety using in vitro and in vivo tests. To achieve a desired gene expression, therapeutic genes must penetrate two barriers: the first barrier is the dense outmost skin layer, the stratum corneum, which prevents therapeutic agents from freely entering the body. The second is the cell membrane, which prohibits large polar or ionic molecules, such as DNA or RNA, from entering the cells. To overcome these barriers during gene therapy, both biochemical and physical approaches have been extensively investigated. Biochemical methods are renowned for their specificity and efficiency, such as the use of viral vectors, liposome-mediated transfer, nanoparticle-assisted delivery, and ligand conjugates. Alternatively, physical methods take advantage of ultrasound, electromotive techniques, thermal effects, microinjection, and gene guns to transfect different cell types. An overview of the approaches and applications of gene transfer is presented in Figure 1A. Each technique provides a unique methodology to improve the efficiency or delivery precision during the gene transfer. For instance, liposomes and nanoparticles ensure the smooth delivery of DNA and RNA to target cells while providing additional functionality of imaging [12,13]. Similarly, physics methods like microinjection directly deliver genetic molecules into the individual cells with high yield; and gene guns enable the transfection of cells that are typically resistant to the biochemical methods.

Electromotive gene transfer methods, as illustrated in Figure 1B–E, have widely adapted to applications ranging from disease treatment to wound healing [1,7,9,11,16,17]. Iontophoresis utilizes a low-intensity current to move nucleic molecules to the target tissue as shown in Figure 1B,C [11,14]. Iontophoresis has a wide range of applications, particularly in the field of medical treatments and diagnostics, including excess sweating treatment and diabetes sensing along with insulin delivery. The primary delivery mechanism is electromigration and electroosmosis and mainly targets the superficial layers of the skin.

Electroporation, another predominant in vivo electromotive gene delivery, has the capability to transfect target cells with high efficacy (Figure 1D,E) [8,10,15]. This technique involves applying short, high-voltage pulses to create temporary nanopores in cell membranes, allowing the introduction of genetic molecules into the target cells. It is a suitable gene therapy strategy for more invasive treatments such as cancer treatments and management of diabetic complications [7,10,15,18,19,20,21,22].

The setup of an electroporation-based in vivo gene transfer system includes two main parts, an electroporator and electrodes. The electroporator is a pulse generator to produces high amplitude with a pulse width ranging from nanoseconds to milliseconds. The electrodes are typically inserted into the target tissue to apply electric pulses for creating nanopores on the cell membranes. Based on the distribution of the electric field, the electroporation can be attributed into two categories: bulk electroporation (BEP) and localized electroporation (LEP). Traditionally, the BEP technique employs metal as electrodes. For in vitro BEP, the commercially available cuvettes use aluminum plates as electrodes. In contrast, in vivo, BEP widely uses needle-type electrodes for both animal models and clinical applications. Recently, with the advancement of nano and microfabrication, the integration of microneedle technology and electroporation brings an innovative approach to this spectrum and has further expanded the potential of gene transfer. This approach incorporates an array of sharp, tiny needles to enhance the gene transfer efficacy in two ways: microneedle-type electrode-based bulk electroporation (MNE-BEP) and hollow microneedle-based localized electroporation (HMN-LEP).

In this comprehensive review, we aim to explore the technical aspects of microneedle-based electroporation techniques for in vivo gene transfer. This review is structured into several key sections. Section 2 provides an overview of the microneedle-based electroporation technique in the domain of in vivo gene transfer. Section 3 delves into the mechanisms of microneedle-type electrode-based BEP and hollow microneedle-based LEP (TNT). We examine the theoretical analysis and representative numerical simulation models of the electroporation process facilitated by MNE-BEP and HMN-LEP. In Section 4, we shift our focus to the fabrication methods of microneedle-type electrodes and TNT silicon chips. This section offers an in-depth look at the manufacturing processes, materials used, and the design considerations that contribute to the functionality and transfection efficacy. Section 5 is dedicated to the diverse applications of MNE-BEP and TNT technologies in the realm of gene transfer. Here, we explore various fields where these techniques have made significant impacts, including tissue reprogramming and wound healing, and we highlight specific cases and studies that demonstrate their practical uses. Finally, Section 6 addresses the challenges and future perspectives of MNE-BEP and TNT. We discuss the current challenges, ongoing research efforts to overcome these limitations, and the potential future directions that this technology could take, paving the way for advancements in medical science and therapeutic interventions.

## 2. Microneedle-Based Electroporation for In Vivo Gene Transfer

### 2.1. Microneedle-Type Electrodes-Based Bulk Electroporation for In Vivo Gene Transfer

Microneedles have been explored in various range of applications, including drug delivery, biosensing, and diagnostics [23,24,25]. It enables the penetration of the dense skin barrier, the stratum corneum layer, to effectively deliver drugs into the epidermis and dermis. However, the delivery efficacy of genes is limited because the cell membrane only allows small, nonpolar, or lipophilic molecules to pass through, serving as a barrier for hydrophilic and polar molecules like DNA and RNA [26]. These genes can enter cells via natural endocytosis, but the transfect efficiency is low. To address this challenge, recent innovations have combined microneedle technology and bulk electroporation, achieving higher transfection efficacy by overcoming both barriers of the stratum corneum and the cellular membrane. This approach demonstrates significant enhancement of gene transfer efficiency in vivo [5,8].

**Figure 2:**
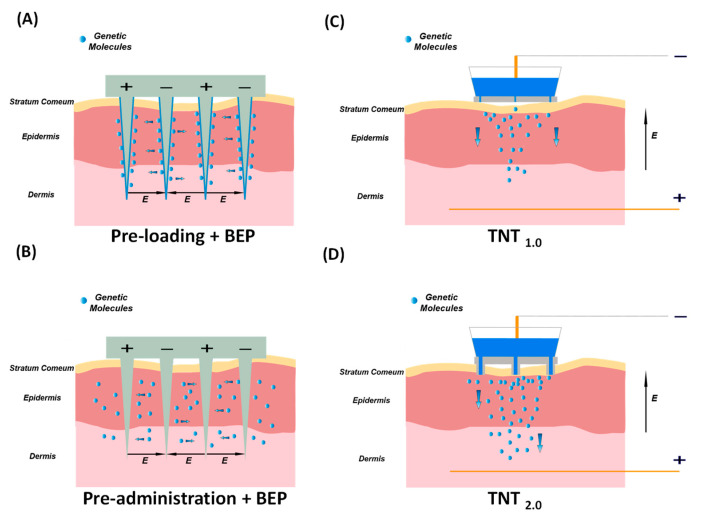
Microneedle-based Electroporation for In Vivo Gene Transfer Systems. Left: Microneedle-type electrodes. (**A**) The genetic molecules are pre-loaded onto the surface of solid microneedles before punching the skin. (**B**) The genetic molecules are pre-injected intradermally, followed by electroporation. Right: TNT-based in vivo gene transfer systems. (**C**) The first generation of TNT_1.0_ chip with nanochannels. (**D**) The second generation of TNT_2.0_ chip features a hollow microneedle array.

The MNE-BEP approach uses conductive microneedles as electrodes to apply electric pulses in vivo as illustrated in Figure 2A,B. The conductive microneedle array can be inserted into the skin to bypass the high resistance of the stratum corneum barrier. In addition, the required voltage compared to the traditional BEP is reduced, due to the smaller distances between electrodes (typically less than 1 mm). In this technique, the generic molecules are coated on the microneedles before the electroporation (Figure 2A or injected into the skin (Figure 2B). Due to the microneedles not reaching the deep dermis, the nerve-rich layer, the pain can be dramatically reduced, and the delivery depth can be precisely controlled with insertion depth. Figure 1D shows an example of delivering COVID-19 DNA vaccination using the MNE-BEP approach [8]. In their experimental setup, six rows of stainless-steel microneedles were mounted on a 3D-printed insulative substrate, with a spacing of 0.9 mm between each row. Before electroporation, a plasmid DNA vaccine was intradermally administered to the skin. Subsequently, the metallic microneedle array was inserted into the injected area, followed by the application of electric pulses provided by a piezoelectric-based electroporator. This method resulted in the successful transfection of target cells within the epidermal layer, leading to an immune response that was observed to be at least an order of magnitude higher compared to traditional intradermal injections without the application of BEP. More details about the mechanism and device fabrication will be reviewed in the following sections.

### 2.2. TNT for In Vivo Gene Transfer

TNT is an innovative in vivo gene transfer technology, that employs the mechanism of HMN-LEP as illustrated in Figure 1E. TNT technique comprises a silicon (Si) chip, metal electrodes, and microsecond electric pulses to directly transfect target cells within the tissue. TNT successfully demonstrated transdermal gene therapy for skin repair [18], tumor regression [19], ischemic stroke recovery [27], and extreme chronic wound healing [20]. The first generation of the TNT_1.0_ chip utilizes the mechanism of nanoelectroporation via nanochannels as illustrated in Figure 1E and Figure 2C. Fibroblast cells of skin in vivo have been successfully reprogrammed into neuronal and endothelial cells by delivering specific gene cocktails in mice models [7,10,21]. The second generation of TNT_2.0_ chips that feature a hollow microneedle array is shown in Figure 2D. This modification is aimed at enhancing the physical contact between the TNT chip and skin to accommodate the nonuniform topography across the skin, thereby improving gene delivery efficiency. The TNT silicon chip exhibits advantages in terms of precise targeting, controlled release, and higher gene transfection, making it an invaluable tool in gene therapy and research. As this technology continues to evolve, it holds great potential for clinical applications in the fields of tissue engineering, regenerative medicine, and wound healing.

## 3. Delivery Mechanisms of Microchip-Based In Vivo Gene Transfer

Electroporation enhances gene transfer via increasing cell membrane permeability. When the cells are exposed to an external pulsed electric field, the nanosized aqueous pores are created across the lipid bilayer, providing channels for the genetic molecules. The mechanism of electroporation has undergone extensive rigorous studies [28,29,30,31,32,33,34,35,36,37], focusing on aspects such as the cellular membrane’s response to external electric fields and the distribution of nanopores on the membrane. Additionally, a significant amount of work has been dedicated to developing mathematical models to simulate cargo transportation at the tissue level. These studies have collectively contributed to the understanding of the electroporation process and provided theoretical insights for designing the devices. In this section, we conduct a review of various representative models that have been developed for in vivo gene transfer, utilizing both bulk electroporation and localized electroporation, particularly TNT silicon chips.

### 3.1. Bulk Electroporation for In Vivo Gene Transfer

Bulk electroporation has been widely used for in vivo gene transfer across various applications from vaccine to disease treatment [2,8,38]. As mentioned in the previous section, this technique can be performed using simple electrodes or advanced microneedle-type electrodes. Based on the cell viability, electroporation can be categorized as reversible and irreversible, each defined by critical thresholds of the electric field. The first threshold represents the starting point of nanopore formation in the cell membrane, enabling the passage of genetic material into cells. The second threshold is the point at which cells cannot recover from electroporation, thereby cell death occurs. The ideal voltage used in electroporation-based gene transfer lies between the first threshold and the second threshold to enable sufficient gene transfer while maintaining cell viability. Therefore, the distribution of the electric field across the target tissue is a primary criterion in the electrode design process. The simulation results can guide the choice of dimension and arrangement of electrodes for optimal transfection outcomes. It should be noted that the critical thresholds for electroporation vary widely based on multiple parameters, including species, cell type, and pulse duration. As such, numerical simulations serve as an essential tool for preliminary estimates of electroporation parameters, aiding in the optimization of electrode design and electroporation protocols without the need for animal testing.

During in vitro bulk electroporation, the electric potential across the cell membrane changes in response to the external electric pulses. This can be explained using the steady-state electrical field distributions with the assumption of a nonconductive spherical membrane [39]. Schwan’s equation is listed below in Equation (1).

(1) $$∆V=\frac{3}{2}ERcos(\theta )$$

where ∆V denotes the induced potential difference across the membrane at an angular position defined by angle θ. And *E* and *R* represent the electric field and the cell radius, respectively. A more comprehensive model that takes the physiological values or the different cell shapes into account can be found in reference [40] with a detailed derivation process.

However, given the complex structure of biological tissues—which may comprise multiple layers with distinct physical characteristics, the numerical simulation with high-resolution meshing is typically required to accurately map electric field distributions. This large-scale meshing consumes high computing resources as well as a long time. Therefore, simplified models are used in numerical simulation for electroporation modeling.

In a simplified mathematical model, the tissues can be viewed as a uniform structure that is electrically homogeneous [41,42] or a combination of circuit components, such as resistors and capacitors [37,43]. The finite element methods are applicable for the simplified uniform structure using the Electric Currents module in commercially available software (such as COMSOL Multiphysics). The simulation is based on the following equations (Equation (2)) [44]:

(2) $$\left\{\begin{array}{c} \nabla \cdot J=Q_{j}\\ J=\sigma E+J_{e}\\ E=-\nabla V \end{array}\right.$$

J denotes the current flow. *Q* and σ represents the electric charge and electric conductivity, respectively. The estimated electric field that is derived from finite element modeling is utilized to evaluate the electroporation with various settings.

**Figure 3:**
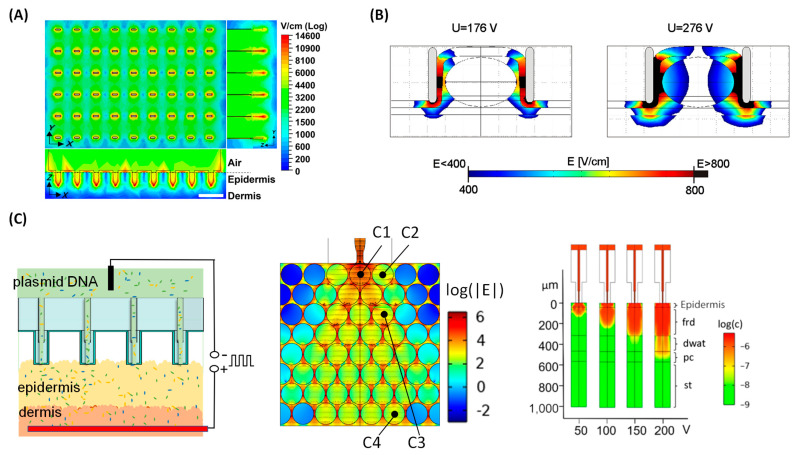
Simulations for Tissue Electroporation. (**A**) Electric filed distribution of BEP with microneedle-type electrodes on a tissue model. The color bar represents electric field strengths. Scale bar: 1 mm. Figure adapted from [8] under terms of the CC-BY license. Copyright, 2021, the Author(s), Published by PNAS. (**B**) Modeling of BEP with bulk electrodes. The tissue is considered as a multi-layer structure. Figure adapted from [45] under terms of the CC-BY 2.0 license. Copyright, 2013, the Author(s). Published by BioMed Central Ltd. (**C**) Numerical simulation of TNT. The left figure shows a schematic view of a TNT process. The skin is modeled as a cell array (middle) or a multi-layer structure (right). Figures adapted from [44] with permission. Copyright Tsinghua University Press and Springer-Verlag GmbH Germany, part of Springer Nature 2021. It is not subject to the open access terms of this article and may not be reproduced without additional permission from the original copyright holder.

Figure 3A shows a simulation result of electric field distribution for the microneedle-type electrode setting [8]. In this skin model, the same resistance is employed for the epidermis and dermis. The observed peak electric fields reach maximal intensities near the microneedle tips but degrade rapidly as the distance from these tips increases. This finding indicates that numerical simulation is a critical tool for understanding the mechanism of electroporation.

An alternative and refined approach employs multi-layer structures with distinct electric properties within each layer [45,46,47] to mimic the composition of tissues, such as skin layers. Figure 3B represents a simulation result of electric field distribution during bulk electroporation to treat a tumor placed between two electrodes [45]. The skin is considered a multi-layer structure, comprised of the stratum corneum, epidermis, dermis, fat layer, and muscular layer, as shown in Figure 3B. The central elliptical structure is a representation of the tumor. In each layer, the electrical properties are the same, but the properties vary distinctly between different layers. The simulated electric field distribution suggests significant differences in the local electric field in the tumor and surrounding environment at different voltages [45]. This numerical calculation enables the estimation of the local electric field intensity at designated target locations. This management is crucial to achieve a uniform electroporation effect and optimize gene transfer effectiveness while minimizing cellular damage in gene therapy.

Instead of solely analyzing electric field distribution, complex models are developed in which both the electric field and gene migration are comprehensively integrated for a better understanding of the gene transfer process [48,49]. The detailed theoretical framework will be discussed in the following subsection using TNT as an example.

### 3.2. Tissue Nanotransfection for In Vivo Gene Transfer

Tissue nanotransfection is specifically designed for localized electroporation of in vivo gene therapy. The initial simulation was mainly focused on the electric field distribution and heat dissipation [10]. Later, it used a comprehensive approach to take into consideration both the membrane permeabilization and the movement of genetic molecules [44]. By including electrophoresis in the model, it provides a method to estimate gene delivery depth and transfected area within the tissue. Those characteristics provide the basis delivery mechanism of the TNT process. The nanopore distribution model is adopted and combined with the diffusion model to simulate the delivery process of genetic molecules from hollow microneedles to the target cells. This model considers dynamic pore evolution in response to time-varied transmembrane voltage (TMV), which consists of two components. The first part is the resting potential difference between the intracellular and extracellular environments. The second aspect is the temporally varying TMV induced by external electric pulses. The quantification of pore number (N) can be effectively addressed using the asymptotic model, which is a simplified version of the Smoluchowski equation by transforming it from a partial differential equation to an ordinary differential equation as shown in Equation (3). The simplification enables solving it numerically in a cost-effective way [33].

(3) $$\frac{dN(t,\theta )}{dt}=\alpha e^{{\left(\frac{V_{m}\left(t,\theta \right)}{V_{ep}}\right)}^{2}}\left(1-\frac{N\left(t,\theta \right)}{N_{0}e^{q{\left(\frac{V_{m}\left(t,\theta \right)}{V_{ep}}\right)}^{2}}}\right),$$

where the *N*, *V_m_* denotes nanopore density and TMV at time *t* and angular position θ, respectively. *N*_0_, $V_{ep},\alpha ,q\;$represent modeling parameters. The asymptotic model assumes that prior to the application of an electric pulse, the cell membrane contains no hydrophilic pores, and the formation of hydrophilic pores starts from an initial radius. The radii ($r_{j})\;$of the pores are also time-varied as described by Equation (4) [44,50]

(4) $$\frac{dr_{j}}{dt}=v\left(r_{j},V_{m},\tau_{eff}\right),\;j=1,2,\ldots M,$$

where v and $\tau_{eff}$ represents the drift velocity of hydrophilic pores, and the effective surface tension of the cell membrane, respectively. Integrating both equations along with the electric field distribution can obtain the nanopore distribution in response to the electric pulse stimuli at the single cell level. However, for modeling the gene transfer process at the tissue level, the diffusion effects must be incorporated into the gene migration simulation. In such instances, the Nernst-Planck equation can serve as a robust tool for simulating the negative-charged genetic molecules’ electrokinetic movements within the tissue as Equation (5) [44,51].

(5) $$\frac{\partial c_{\pm }}{\partial t}=\nabla \cdot (D_{\pm }\nabla c_{\pm }\pm D_{\pm }\frac{z_{\pm }e}{k_{B}T}c_{\pm }\nabla \varphi -c_{\pm }u)$$

where D, u, represents the diffusivity coefficients of positive and negative species, the flow field, respectively. c and e are the concentration and elementary charge.

An overview of the simulation model of TNT is shown in Figure 3C—(left), and the middle one illustrates a cell-array structure used for modeling the tissue [44]. The cell-array model primarily evaluates the cellular-level response to electric pulses. The electric pulse is applied between the top and bottom electrodes as illustrated in Figure 3C—(left). The cell array model (Figure 3C—(middle)) is used to simulate the transfection process on a mice skin model. The electric field distribution within the tissue is numerically computed upon the application of a series of short pulses. Subsequently, this data is utilized as the time-varying electric field in the nanopore formation and gene migration. The gene transfections are observed at various locations (cells C1 to C4). The electric filed distribution across the cell array is simulated and presented in the middle section of Figure 3C.

An alternative tissue modeling strategy is presented in Figure 3C (right). This structure has been extensively used in skin models due to its multi-layered characteristics that better represent the real skin. The delivery distance is determined by the vertical depth reached by the genes in the tissue. The multi-stack layer model provides a larger scope of gene delivery process more quickly compared to the cell-array model which requires extensive calculation time, due to limited computational resources.

Mathematical modeling is an essential strategy to investigate the tissue transfection mechanism. Consequently, it is used for device optimization to boost gene transfer efficiency. However, at the current stage, the mechanism of in vivo electroporation is not fully understood, primarily due to the complex biological structures and the various physical effects involved. The electroporation-based in vivo gene transfer process includes not only electroporation but also electrophoresis, thermal effects, and other biochemical responses, forming a highly dynamic and complicated system. Fortunately, with the rapid development of machine learning technologies, we anticipate the integration of artificial intelligence and multiphysics simulation will yield faster and more accurate modeling, potentially overcoming the current limitations. This advancement can enable us to better understand the mechanism of electroporation-based gene transfer in vivo.

## 4. Fabrication of Microneedle Chips for Electroporation-Based In Vivo Gene Transfer

As mentioned above, compared to conventional electrodes, the microneedle-type electrodes exhibit better pain management and lower voltage requirements due to the microscale needle dimensions. To manufacture microneedle-type electrode devices with precise patterning and high yields, sophisticated manufacturing techniques are often utilized. Some of these techniques include laser-cutting, metallization, and semiconductor processes. In this section, we focus on the review of various device fabrication methods to provide insights into the development of microneedle-based chips for in vivo gene transfer.

### 4.1. Fabrication of Microneedle-Type Electrodes-Based BEP Chips

Microneedles are designed to penetrate the stratum corneum, the outmost layer of the skin, which enhances transdermal drug delivery [52,53,54]. These microneedles do not reach the nerve cells found in the dermis, providing a painless or minimally painful experience. The microneedles can be categorized into five classes: solid, coated, hollow, dissolvable, and hydrogel-forming [55,56,57]. Solid microneedles work by creating open channels in the skin, followed by topical delivery of drugs. In contrast, coated microneedles deliver the drugs by which the drugs are pre-loaded on the microneedle surface. Hollow microneedles enable controlled drug release like traditional hypodermic needles dose. Dissolvable microneedles release encapsulated drugs over time since it is water soluble. Interestingly, hydrogel-forming microneedles not only deliver drugs but also have the capability of extracting interstitial fluid from the skin for monitoring purposes, due to the swelling nature of the hydrogel [25,58].

**Figure 4:**
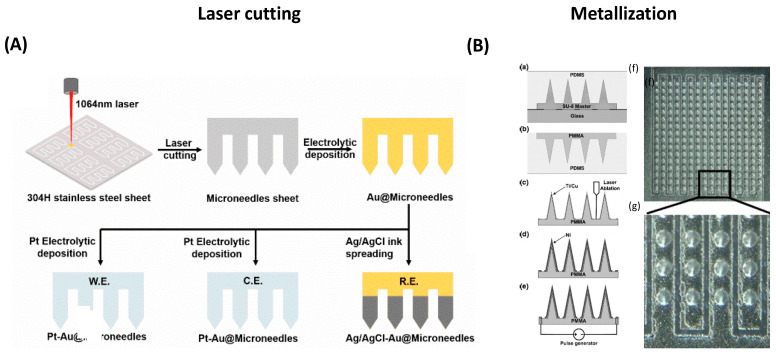
Fabrication Processes for Microneedle-based Electroporation Devices. (**A**) Fabricating stainless steel microneedles with laser cutting. Adapted from [61] under terms of the CC-BY license. Copyright, 2022 by the Author(s), Published by Micromachines. MDPI. (**B**) Fabrication process of conductive microneedles using metallization technique. Figure adapted from [4] with permission. Published by Springer Nature Copyright 2009, Springer Science Business Media, LLC. It is not subject to the open access terms of this article and may not be reproduced without additional permission from the original copyright holder.

The representative arrangement of the MNE-BEP device is illustrated in Figure 4A,B. The electrodes are in an alternative pattern of anodes and cathodes to facilitate the application of an electric pulse between them.

Stainless steel is a predominant material for conductive microneedles due to its low cost, superior mechanical strength, and excellent machinability. It is also an FDA-approved material, widely used in medical applications such as hypodermal syringes and surgical instruments. These characteristics allow precise manufacturing of microneedles, including sharp tips and streamlined shapes, for effective skin penetration without the risk of deformation or breakage, which may cause extra pain or wound.

Laser cutting is a common technique used in both laboratories and industries to make metallic microneedles [59,60]. It provides precise control over microneedle dimensions, a critical factor for ensuring consistent and effective transdermal penetration and gene delivery. This process employs a high-intensity laser beam to precisely cut a thin metal sheet to form the microneedle shape as illustrated in Figure 4A [61]. The layouts of microneedles are predetermined using computer-aid design software. When the laser beam hits the metal surfaces following the design layout, rapidly increase the temperature to achieve material separation via melting or vaporization. After the microneedles are fabricated from the metal sheet, additional processing steps, including cleaning, electropolishing, extra layer coating, and drying, are required to enhance the needle quality and functionality.

Another approach for fabricating microneedle-type electrodes is metallization of non-metallic microneedles [4,5]. Various metallization methodologies, such as thermal evaporation, electron-beam evaporation, sputtering, and electroplating, are applicable for the deposition of metals with desired thicknesses onto prefabricated microneedles. Figure 4B illustrates an MNE-BEP chip through the metallization processes on a polymer microneedle array [4,5]. Initially, the PMMA (Polymethyl Methacrylate) microneedles are fabricated using a PDMS (Polydimethylsiloxane) mold as a template. Subsequently, these microneedles were coated with thin metal layers of Titanium (Ti) and Copper (Cu) utilizing a sputtering system. The next step involved patterning these coated metal layers through laser ablation. Finally, a 20-μm thick Nickel (Ni) was electroplated to complete the electrode array for the BEP application. The chip has demonstrated good performance on human skin without experiencing any mechanical failures. Its effectiveness was demonstrated through the successful transfection of red blood cells and human prostate cancer cells, highlighting its promising capabilities for use in clinical applications.

### 4.2. Fabrication of TNT Si Chips

**Figure 5:**
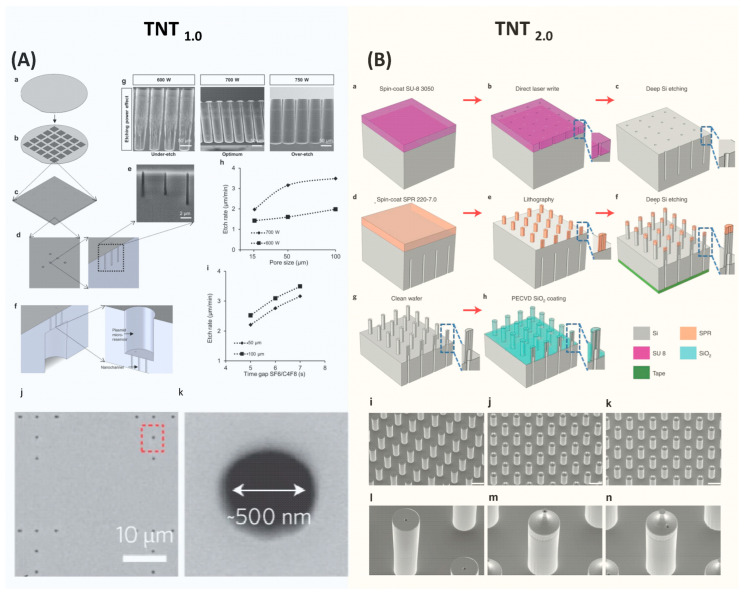
Semiconductor Process for Fabricating TNT Si Chip. (**A**) The device fabrication process of TNT_1.0_ chip. Figures adapted from [10] with permission. Copyright 2017, Springer Nature Limited. Published by Nature Nanotechnology, Springer Nature Limited. (**B**) The device fabrication process of TNT_2.0_ Si chip. Figures adapted from [62] with permission. Copyright 2021, The Author(s), Published by Nature Protocols, Springer Nature Limited. Figure 5 is not subject to the open access terms of this article and may not be reproduced without additional permission from the original copyright holder.

Unlike the MNE-BEP, the microneedles in TNT are not used as electrodes. The TNT setup is comprised of three primary hardware components: a TNT silicon chip, electrodes, and an electroporator. The pivotal component among these is the TNT silicon chip, a hollow channel array, especially a hollow microneedle array fabricated on a Si substrate (Figure 5). In the TNT process, plasmid DNA is delivered to the tissue through these hollow channels with electrophoretic force [10,44,62].

Two types of TNT chips have been developed [10,62]. The initial version of the TNT_1.0_ chip features nanochannels on a flat surface [10]. Subsequent enhancements led to the development of TNT_2.0_, which is a hollow microneedle array [62]. This design aimed to improve the contact between the chip and the skin surface, thereby enhancing delivery efficiency. In addition, the bore size increased from several hundred nanometers to a few microns. Both TNT chips were fabricated using Si because of their superior mechanical properties, favorable biocompatibility, and mass producibility using the standard semiconductor process.

The fabrication process of Si microneedle arrays has been optimized for particular shapes, inner structures, materials, and functionalities by combining CMOS and MEMS technologies. Dry and wet etching methods are the most common techniques to fabricate microneedles, but molding and electroplating are also common too [63,64,65,66,67].

TNT silicon chips are fabricated using the standard semiconductor process, primarily involving dry etching and optical lithography processes [10,62]. The brief fabrication process of TNT_1.0_ is shown in Figure 5A [10]. It starts with a 4-inch double-side-polished Si wafer with a thickness of ~200 µm. First, a thin photoresist was coated on the front side of the wafer, followed by patterning a nanohole array with a diameter of ~500 nm using optical lithography. These nanohole patterns were then etched into the Si wafer using a plasma etcher, forming nanochannels with ~10 µm deep. After removing the residue photoresist, the wafer was then flipped and a microhole array was patterned on the backside and etched to form micro reservoirs. These microreservoirs are directly connected to the nanochannels, enabling the unimpeded flow of gene solutions from the microreservoirs to the nanochannels, and eventually entering tissues via the electrophoretic force during the TNT process. The Si etching recipes were optimized to deliver the desired etching profiles and qualities. In the final step of the process, a thin silicon nitride film was deposited over the TNT chip to electrically insulate it.

The fabrication approach employed for TNT_2.0_ is similar to TNT_1.0_, yet it is more complicated as detailed in Figure 5B [62]. The process begins with spin coating a thick photoresist on one side of a silicon wafer, which was then patterned to define a microhole array using optical lithography. Subsequently, this pattern was etched to a specific depth using deep Si etching to form micro reservoirs. Next, a photoresist was spin-coated on the opposite side of the wafer and patterned to outline hollow microneedles with precise alignment. This is a critical step to ensure that the hollow microchannels and microreservoirs are well aligned. Employing the etching process again, the hollow microchannels were etched until they connected to the previously etched backside micro reservoirs.

The fabrication of hollow-type Si microneedle arrays is quite challenging, and the key process is to develop a high-aspect ratio of Si etching. A specialized etching technique of deep Si etching called the Bosch process, plays a crucial role [68]. This process alternates between isotropic SF_6_ etching and C_4_F_8_ passivation, enabling rapid Si etching with the vertical sidewalls. A distinctive feature of the Bosch process is its high etching selectivity [69]. Through fine-tuning of process parameters, the Bosch process enables the etching of Si to create deep trenches using relatively thin layers of photoresist or silicon dioxide as etching masks. The etching selectivity can reach over 100 with careful optimization of etching parameters. This high selectivity is particularly useful in the fabrication of complex 3D structures. However, in the standard two-step Bosch process, the achievable aspect ratio is typically 15:1 [69]. This restriction primarily results from the increased difficulty in the diffusion of etching gas into the deep and narrow microstructures and the re-deposition of etching byproducts at the bottom of the etched areas, which prevents further etching [70,71,72].

The aspect ratio of the TNT Si chips is over 15:1. To fabricate this high aspect ratio structure, a three-step Bosch process is employed [62,73]. This Bosch process incorporates passivation, clearing (involving the removal of passivated polymers using active argon), and etching. As a result, a high aspect ratio of over 30:1 can be achieved. In addition, using ramped process parameters, such as modulation of plasma energy, etching time, and pressure over time, demonstrated improved aspect ratios [74].

The diameters of hollow channels significantly impact the distribution of the electric field across the tissue, which is one of the factors influencing the delivery depth and the amount of transferring genetic molecules [44]. Therefore, after initial fabrication, the tailoring of the bore sizes of hollow needles is necessitated with thermal oxidation, chemical vapor deposition (CVD), or plasma-enhanced chemical vapor deposition (PECVD). Deposited materials, such as silicon dioxide or silicon nitride, effectively reduce the bore sizes to the target sizes. Additionally, these coated layers serve as an insulator between the skin and the TNT Si chip, ensuring the electric field only passes through the hollow microchannels to generate a localized electric field.

The prospective advancements in the domain of TNT device fabrication technology will focus on further enhancing the gene transfer efficacy in various circumstances such as deep tissue. This will involve refining the microneedle with shaper tips to ensure penetrates the stratum corneum layer to transfect the deeper tissue. Additionally, the focus will be placed on the system-level integration, which assembles various engineering components such as an electroporator and electrodes into the Si chip to minimize the footprint of setup.

Furthermore, the scalability of TNT manufacturing processes will be a critical task when transitioning from laboratory-scale production to large-volume manufacturing. This shift is vital for meeting the demands of clinical applications, ensuring cost-effective and wide accessibility for patients.

## 5. Applications of Microneedle-Based Electroporation Gene Transfer

In this section, we will explore the diverse range of applications utilizing electroporation-based in vivo gene transfer, particularly focusing on the essential role of the TNT technique in gene therapy.

The integration of microneedle technology and electroporation opens a new era in the field of gene transfer. The unique strengths of both microscale resolution devices and widely suitable electroporation benefit a wide range of applications across diverse fields. The development of MNE-BEP has significantly enhanced the aspects of pain management, reduced voltage, and delivery efficacy. Furthermore, the clinical studies showed a promising future in cancer treatment [9,75], skin disease treatment [41], and DNA vaccination [4,8].

TNT takes additional measures to use a localized electric field, allowing for precise targeting, controllable delivery, and excellent transfection efficacy. For its application, the TNT technique can be classified into in vivo tissue reprogramming and non-reprogramming types based on whether the gene transfer induces cell-type changes in the tissues. In the treatment of diseases and repair of damaged tissue, patients often lose some capability to generate specific functional cells. This deficiency significantly affects the treatment outcomes and prevents the healing process. To improve this situation, in vivo tissue reprogramming via TNT presents a groundbreaking method for directly converting a type of cell (such as fibroblast) into desired functional cells in vivo [21]. This innovative approach bypasses the intermediate stem cell stage to increase efficiency and reduce the risk of tumorigenesis [10]. In the current research, the TNT silicon chip-based cell reprogramming has been demonstrated in the field of wound healing, diabetes, ischemic diseases, and cancer treatment as illustrated in Table 1. Non-reprogramming TNT applications, on the other hand, result in no change in the cell type while increasing the gene expression efficacy in the disease treatment process or exosome labeling.

In the realm of wound healing, the application of electroporation-based in vivo gene transfer has been proven to be a transformative approach. This direct method yields a high delivery efficiency of therapeutic genes into target cells at the wound site within a second. This approach can be especially beneficial for chronic wounds, a condition often resistant to traditional healing methods [20], and counteracting the underlying issues in these wounds, such as inflammation [76,80] or insufficient blood circulation [78]. This method can effectively treat patients with complex wounds.

Microchip-electroporation-based in vivo gene transfer also shows great promise in the field of cancer treatment. Microchip technology enables the precise delivery of cancer-fighting genes directly into tumor cells or surrounding tissues. Unlike conventional treatments that often affect both healthy and cancerous cells, TNT allows for the delivery of therapeutic genes in a highly localized manner, ensuring maximum impact on the tumor while minimizing side effects. Except for the in vivo gene delivery methods [9,17,19,79] mentioned in this review, irreversible electroporation is also widely used to induce tumor cell death for cancer treatment [81].

Effective treatment for diabetic ischemic limbs is crucial in diabetes management, as it significantly impacts the patient’s daily life. The electroporation-based gene transfer can notably improve the ischemic conditions in diabetic mice using TNT [22].

It is worth pointing out that the potential applications for the TNT Si chip are far greater than its current scope of use. We will discuss it in detail in the next section about limitations and future perspectives of TNT silicon nanochip.

## 6. Challenge and Future Prospects

The utilization of the microneedle-electroporation-based in vivo gene transfer facilitates a widely suitable strategy to target various cell types, providing effective gene therapy for treating diseases. Nonetheless, it also faces several challenges, including the need for specific protocol optimization, the bulk size of the pulse generator, and concerns about cell viability due to high amplitude electrical pulses. In this section, we will discuss the limitations and challenges inherent in electroporation-based in vivo gene transfer techniques and propose potential solutions.

### 6.1. Industrialization

TNT Si chips have been successfully fabricated in college-level nanofabrication facilities. The process involves many steps, including wafer cleaning, photoresist coating, optical lithography, deep Si etching, CVD deposition, and wafer dicing. Under optimal conditions, it takes several days to process a 4-inch wafer, producing up to 16 TNT chips, assuming a 100% yield. However, several process issues, such as etching uniformity and particles, result in a reduced yield of functional TNT chips. Specifically, non-uniform Si etching can obstruct the hollow channels in certain areas of the chip due to insufficient Si etching, blocking the free flow of gene solutions. Furthermore, the introduction of unwanted particles from tweezers, glassware, chambers, dicing, and the environment poses additional risks, such as potential tissue infections. While these issues can be improved to a certain degree, the limitations of a college-based nanofabrication facility remain evident compared to the dedicated semiconductor foundries.

The TNT process does not involve any metallization process, and is capable of fabricating on 300 mm wafers, dramatically increasing the production volume compared to 100 mm wafers employed in academic settings. Furthermore, high aspect ratio etching technologies are well established and are employed in current productions, such as through-silicon-via (TSV) in 3D LSI integration and 3D capacitors in DRAM [82,83]. Therefore, TNT Si chips can be manufactured in semiconductor foundries using the standard semiconductor process, enabling high-volume mass production for potential future commercialization.

### 6.2. Electroporation Protocol Optimization

As discussed in Section 3, the electric field required in electroporation has an optimal parameter set based on the type of animals and cells due to the various physical properties such as tissue structure, conductivity, and porosity. Those properties affect both the electric field distribution across the tissue and the diffusion velocities of the genes. Therefore, to achieve optimal outcomes, it is crucial to optimize the protocols including pulse waveform, pulse duration, and device dimension. Currently, this protocol optimization is mainly conducted through a combination of in vitro and in vivo animal models. With the advancement in recent artificial intelligence, this process could be improved by harnessing the power of machine learning-based optimization [17,84,85,86]. The integration of machine learning and multiphysics-based numerical simulations can enable the rapid and precise refinement of pulse parameters and device design. Importantly, this approach could play a crucial role in significantly reducing the reliance on animal models during the protocol optimization stage and align with the ethical research practice.

### 6.3. Bulk Electroporation System

Another significant challenge of electroporation-based gene therapy lies in the current bulky setup. The high-voltage pulse generators are typically large and heavy, which limits TNT use in clinical settings and urgent scenarios. Some studies show promising potential in developing portable electroporators [8,87,88,89,90]. The strategies include piezoelectric-based and tunable circuit-based compact pulse generators. This progress indicates a promising trajectory for the evolution of electroporation-based gene transfer technology.

### 6.4. Clinical Translational

As mentioned in the previous section, the electroporation technique can be categorized into reversible electroporation and irreversible electroporation based on the cell viability. The irreversible electroporation method is widely used in cancer treatment to ablate cells in tumor tissue via strong electric fields. This technique has gained significant attention and has been applied in clinical trials for treating diseases in the liver, pancreas, skin, and kidney [91]. In contrast, the reversible electroporation for gene therapy remains under active research to address safety concerns for wide employment in clinical trials.

To induce nanopore formation in cell membranes, electroporation employs a high-amplitude electric pulse, which exceeds the safety threshold recommended for human exposure. Importantly, such approach as presented by TNT enables in vivo tissue reprogramming [10,21,62]. The excessively high voltage raises concerns about potential cell damage during the gene transfer process. Unintended cell injury carries the risk of reducing gene transfer efficiency and inducing adverse side effects by impacting non-target tissues. In the pursuit of making electroporation-based gene treatments in clinical applications, the primary objective is to lower the operating voltage to a safe threshold while providing high-precision control of localized electroporation. The voltage reduction can be achieved through further optimizing the parameters such as electric pulses, durations, and device dimensions. Additionally, integrating electroporation with other physical techniques may also contribute to lowering voltage, and enhancing overall safety and feasibility. The challenge of exceptional accuracy in electroporation localization may be overcome through further advancements in devices and setup optimization via nanoscale techniques.

## 7. Conclusions

This review has highlighted the mechanism and fabrication techniques in microneedle-based electroporation for in vivo gene transfer, particularly the innovative tissue nanotransfection technology which has proven to be effective in achieving in vivo tissue reprogramming. The application of this technology spans various other therapeutic areas including tumor regression and wound healing. However, challenges such as optimization of electroporation protocol and system still are ongoing subjects for development for practical clinical applications. Interdisciplinary collaboration will be key in advancing this technique including artificial intelligence, multiphysics-based mathematical modeling, and nanotechnology. Future efforts should concentrate on improving increasing gene delivery and expression efficiency.

## Figures and Tables

**Table 1:** Summary of TNT-Silicon-Nanochip-based Gene Transfer Applications.

Applications	Target	Function of TNT	Reagent	Ref
Wound Healing	Mechanism	Fibroblast state change	Anti-miR-200b	[21]
	Rescue muscle loss	Fibroblast into myogenic cells	*MyoD*	[16]
	Rescue Necrotizing Tissues	Fibroblast into neuronal Cells and Fibroblast into endothelial cells	*Ascl1, Brn2* and *Myt1l* *Etv2*, *Foxc2* and *Fli1*	[10]
	Wound Closure	Significant Acceleration in wound recovery	LNA-anti-pan-miR-29 [18], *Etv2*, *Foxc2* and *Fli1* [76]	[18,76]
	Exosome	Exosome labeling for wound mechanism study	CD9, CD63 and CD81	[15,77]
Diabetes	Management of cutaneous Diabetic Polyneuropathy	Fibroblast into neuronal cells	*Ascl1*, *Brn2* and *Myt1l*	[7]
	Diabetic Ischemic Limb Rescue	effective in limb rescue	EndothelialPLCγ *2*	[22]
Ischemic Diseases	Ischemic Tissue	Fibroblast into vasculogenic cells	*Etv2, Foxc2* and *Fli1*	[27,78]
Cancer	Breast Cancer	Decrease in Tumor Growth	*EV* with *ICAM-1*+miR-146a and *Glut1* [79]	[79]
	Tumor	Tumor regression	tumor-originating EV-borne angio-miR [19]	[19]

## Data Availability

Not applicable.
